# MRI study of medial meniscus degeneration of osteoarthritic knees with or without posterior root tear

**DOI:** 10.1186/s40634-022-00474-y

**Published:** 2022-04-29

**Authors:** Kosuke Hisashi, Takeshi Muneta, Yuji Kohno, Mana Sasaki, Junya Yamazaki, Haruhisa Hayashi, Hideyuki Koga, Toshiyuki Morito

**Affiliations:** 1 Hachioji-Higashi Orthopedic Clinic and Akishima Orthopedic Clinic, Joint Surgery Institute, 36-1 Takakura-machi, Hachioji-shi, Tokyo, 192-0033 Japan; 2 Department of Joint Surgery and Sports Medicine, Tokyo Medical and Dental University, Tokyo, Japan

**Keywords:** Knee osteoarthritis, MRI, MMPRT, Meniscus degeneration, Meniscus extrusion, Tibial slope

## Abstract

**Purpose:**

The purpose of this study was to compare the medial meniscus (MM) degeneration, meniscus extrusion, and tibial joint inclination by using MRI to consider the pathogenesis of posterior root tear (PRT) in medial-type knee osteoarthritis (KOA) both with and without medial meniscus posterior root tear (MMPRT).

**Methods:**

This study used open MRI with flexion sagittal view and included 324 medial-type osteoarthritic knees with a Kellegren–Lawrence grade of 2 or less. Following the exclusion process, 151 knees were selected for MRI analysis. MM degeneration grading was performed according to Jerosch by 5 degrees of 0–4 in four different portions from anterior to posterior. MM medial extrusion (MMME), MM posterior extrusion (MMPE), medial tibial medial slope (MTMS), and medial tibial posterior slope (MTPS) were measured according to previous studies.

**Results:**

MM degeneration in the anterior portion to MCL averaged 1.72 ± 0.67 in the PRT group (*n* = 48) and 1.40 ± 0.78 in the non-PRT group (*n* = 103). The degeneration grade was statistically higher in the PRT group than in the non-PRT group (*p* = 0.050). There was no difference in MM degeneration in the other three portions. MMME averaged 4.02 ± 1.12 mm in the PRT group and 3.11 ± 1.11 mm in the non-PRT group. MMPE averaged 4.22 ± 0.87 mm in the PRT group and 2.83 ± 1.12 mm in the non-PRT group. Both MMME and MMPE in the PRT group were statistically larger than those in the non-PRT group (*p* < 0.001). There was no difference in MTMS between the two groups. MTPS averaged 6.34 ± 2.25° in the PRT group and 5.28 ± 2.23° in the non-PRT group. The MTPS of the PRT group was statistically larger than that of the non-PRT group (*p* = 0.007).

**Conclusion:**

The severity of MM degeneration, extrusion of MM, and degree of tibial slope were compared between medial-type KOA with and without PRT using an open MRI. MM degeneration was more severe anteriorly in the PRT group. The PRT group showed larger MMME and MMPE with greater MTPS.

**Level of evidence:**

III. Retrospective cohort study.

## Introduction

A painful pop is reportedly the typical onset sign of a posterior root tear (PRT) of the medial meniscus (MM). A patient experiences a painful pop during daily activities, such as walking down stairs and standing up. Typically, a medial-type osteoarthritic knee worsens with painful symptoms despite the efforts of conservative treatment. The pathomechanism of the progress of medial-type knee osteoarthritis (OA) includes the deterioration of hoop stress of the medial meniscus (MM) due to a medial meniscus posterior root tear (MMPRT), which increases loading on the articular surface during knee activities, such as standing and sitting, going up and down stairs, and deep sitting.

Allaire et al. reported that MMPRT increases loading on the femorotibial joint (FTJ), the same as in total medial meniscectomy [1]. Another study indicated the increase of maximum contact stress of FTJ from 3841 to 5084 kPa, causing the rapid progression of medial joint degeneration [2]. MMPRT has also been frequently recognised in spontaneous osteonecrosis of the knee (SONK) [3].

Regarding the problems of MMPRT, LaPrade et al. [4] reported that the pull-out repair of MMPRT helps to prevent the progression of knee OA. The outcome of the repair of MMPRT varies among the reports, with no clear consensus regarding its efficacy. Short-term outcomes of the pull-out repair have been favourable [5, 6]. Moon et al. [7] reported that the pull-out repair within less than 13 weeks of the PRT onset prevented MMME in a study investigating the correlation between outcomes and time to surgery. A meta-analysis by Krivicich et al. [8] revealed that the repair prevented the progression of knee OA and significantly decreased the overall need for knee arthroplasty. They recommended the repair of MMPRT in an osteoarthritic knee if the OA grade is not severe. Another meta-analysis by Chung et al. [9] suggested that the MMPRT repair could not prevent the extrusion of MM and the progression of radiographic osteoarthritic changes. Wan et al. [10] reported in the meta-analysis that the Lysholm score and IKDC scale improved following the MMPRT repair, but the repair could not stop the progression of radiographic deterioration of knee OA by Kellegren–Lawrence (KL) grade. The effects of MM degenerative change and lower limb alignment in the MMPRT repair have not yet been fully analysed and understood.

In a rat model after collagen injection, Utomo et al. [11] reported that MM extrusion was evident a day after collagenase injection, and the MM degenerative change progressed with time compared to the control group. Marino et al. [12] reported the existence of MM degeneration in medial-type knee OA; however, the MM degenerative change in medial-type knee OA with MMRPT is not sufficiently understood.

Park et al. [13] reported that the intrasubstance degeneration of MM horizontal cleavage tears in young patients is associated with increased joint line obliquity in the coronal plane. Hiranaka et al. [14] reported that steep medial tibial slope is associated with bilateral MMPRT. The relationship between tibial surface morphology and MM degeneration has not yet been well investigated.

The purpose of the current MRI study was to compare MM degeneration, MM extrusion, and tibial surface morphology between medial-type knee OA with PRT and without PRT. The hypothesis of the study was that MM degeneration is less progressed, and tibial surface slope is steeper in knee OA with PRT than without PRT. The clinical relevance is that the results of this MRI study, evaluating MM extrusions, MM degeneration and tibial slopes generally, will suggest proper treatment strategy for each patient of medial-type knee OA with the PRT.

## Materials and methods

**Fig. 1:**
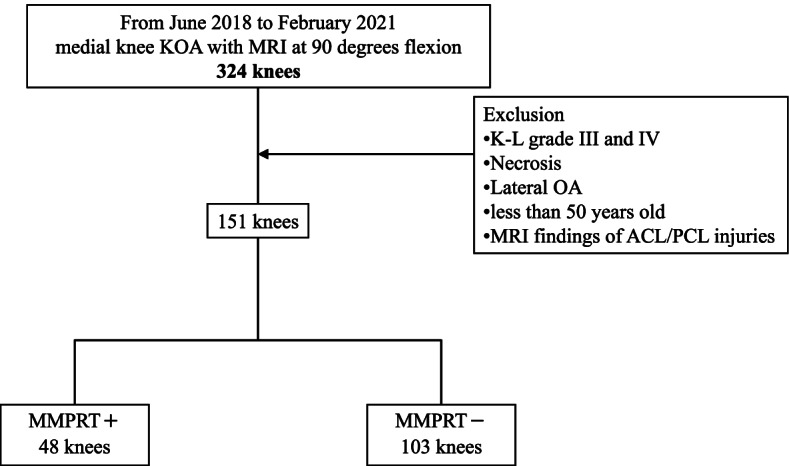
Flow chart of material selection of this study with inclusion and exclusion criteria

A total of 324 medial-type knees with a radiologic KL OA grade of 2 or less at extension and with MRI at extension and the flexion position were retrospectively reviewed in this study. The exclusion criteria were knee OA with KL grades 3 and 4, solitary osteonecrosis, latera-type OA, patient age of 50 years or younger, and previous knee surgeries. The patient with MRI findings of cruciate ligament injury was also excluded from the study (Fig. 1). The diagnosis of PRT has been done according to the positive vertical linear defect and/or positive ghost meniscal sign [15, 16]. In the present study, there were no differences between the PRT group (*n* = 48) and non-PRT group (*n* = 103) with regard to sex, KL OA grade, and age (Table 1).

**Table 1:** Gender, age and osteoarthritic grade for each group

Variables	MMPRT + (48 knees)	MMPRT- (103 knees)	*p*-value
Average age (± SD)	67.9 ± 8.2	65.8 ± 8.3	0.153
Males/ Females	13/35	29/73	0.796
Kellgren-Lawrence Grade			
I	18	36	0.761
II	30	67	

MMPRT: medial meniscus posterior root tear, ns non-significant

The *p*-value determined by Student’s t test was presented up to three decimal places

The study was approved by the institutional review board of the Joint Surgery Institute (No. 2021–01) and conducted according to the Declaration of Helsinki.

### Magnetic resonance image (MRI) conditions

An open MRI of 0.3 Tesla (AIRIS Vento, Hitachi Healthcare, Tokyo, Japan) was used for grading and measurements. Each knee was set at 10 degrees flexion and 90 degrees flexion in a non-weight bearing neutral rotation condition with a specialised knee coil. Standard protocol was as follows: T2-weighted images with 90 degrees pulse (TR: 4000 ms, TE: 95 ms) and proton density images (TR: 2000 ms, TE: 14.8 ms); flip angle 90 degrees; slice thickness of 3.5 mm with a gap of 0.5 mm; FOV of 200; and acquired matrix size of 288 (phase) × 224 (frequency).

### Evaluation parameters

Each MM degeneration grade was assessed in four portions—that is, the anterior to MCL, mid-MCL, posterior to the MCL, and mid-posterior portions. The medial meniscus medial extrusion (MMME) was measured in the coronal view at 10 degrees flexion. The medial meniscus posterior extrusion (MMPE) was measured in the sagittal view at 90 degrees flexion. In addition, the medial tibial medial slope (MTMS) and medial tibial posterior slope (MTPS) were measured in the coronal and sagittal views, respectively.

**Fig. 2:**
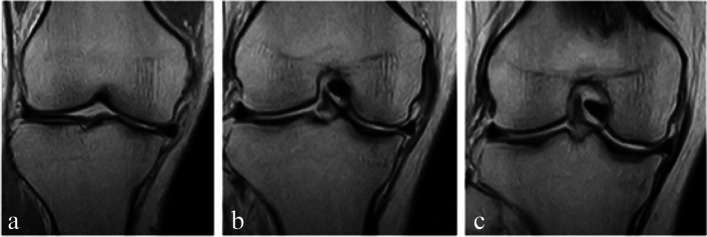
Determination of the slice of medial 3 portions used for degeneration grading. **a**: Slice just anterior to the whole MCL image; **b**: Slice of the longest image of the MCL; **c**: Slice just posterior to the whole MCL image

**Fig. 3:**
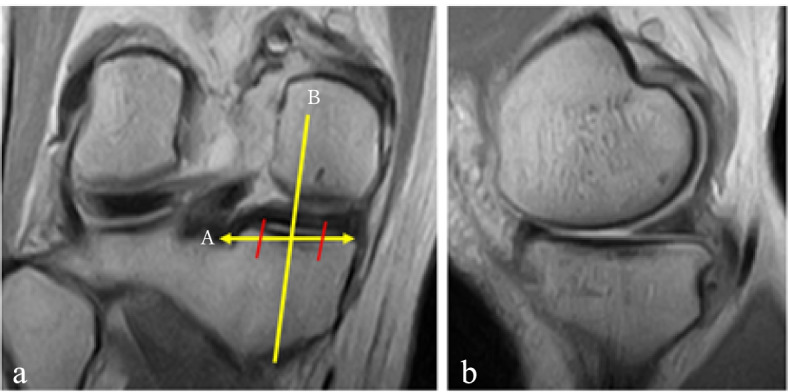
Determination of the mid-posterior slice of MM posterior segment. **a**: Middle slice of the posterior segment in coronal image; **b**: Image of mid-posterior MM slice in sagittal image; **A**: Longest image of MM posterior segment; **B**: Middle slice of MM posterior segment

The meniscus degeneration was graded at the anterior segment next to the MCL, the middle MCL (longest position), the posterior segment next to the MCL (Fig. 2), and the middle of the longest posterior segment (Fig. 3). The meniscus degeneration grading in MRI was determined according to Jerosch et al. [17] by 5 grades from 0 to 4: in grade 0, the meniscus body shows generally low density; in grade 1, a round high-density image is found in the centre of the body; in grade 2, a linear or wedge-shaped high-density image does not reach the meniscus surface; in grade 3, a linear or wedge-shaped high-density image reaches the meniscus surface; in grade 4, a torn or segmented image reaches the meniscus surface.

The MMME was assessed according to Yao et al. [18] in the middle segment of the MM in the coronal view at 10 degrees flexion. The distance from peripheral meniscus edge to the original lateral tibial edge excluding osteophyte was measured in millimetres as MMME. The MMPE was assessed according to Masuda et al. [19] in the posterior segment of the MM in the sagittal view at 90 degrees flexion. The distance from peripheral edge to original posterior tibial edge excluding osteophyte was measured in millimetre as MMPE.

Then, the correlation between the degree of MM degeneration and the medial and posterior extrusion was evaluated for knees without PRT, knees with PRT, and all knees. The degree of MM degeneration of each knee was represented by summing the degeneration score in the four portions.

**Fig. 4:**
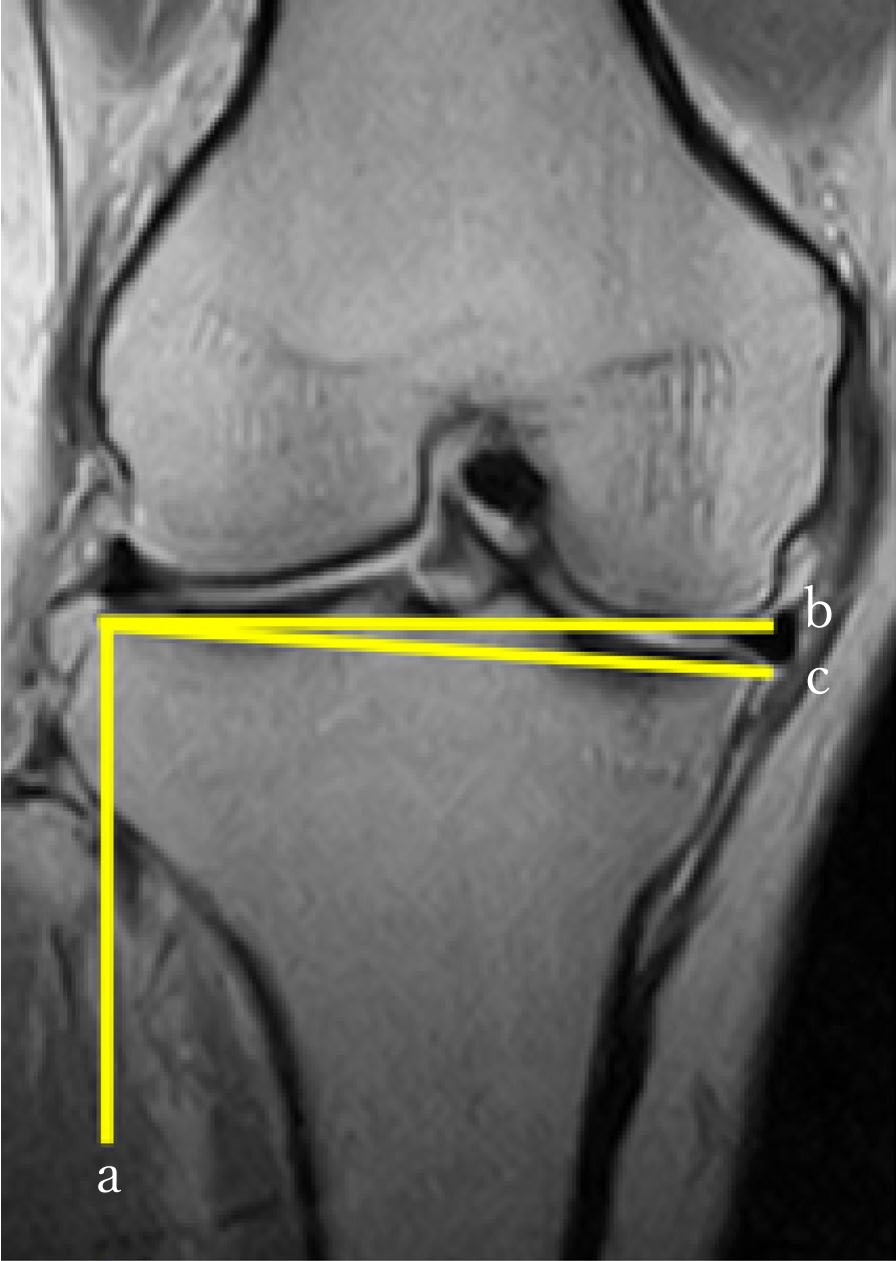
Measurement of medial tibial medial slope (MTMS). **a**: Parallel line of the diaphyseal tibial axis in the coronal slice determined by an axial view of the tibial plateau; **b**: Perpendicular line to the line **a** from the lateral-most point of the tibial joint surface; **c**: Line connecting the lateral-most and medial-most points; MTMS was determined as the angle between lines **b** and **c**

**Fig. 5:**
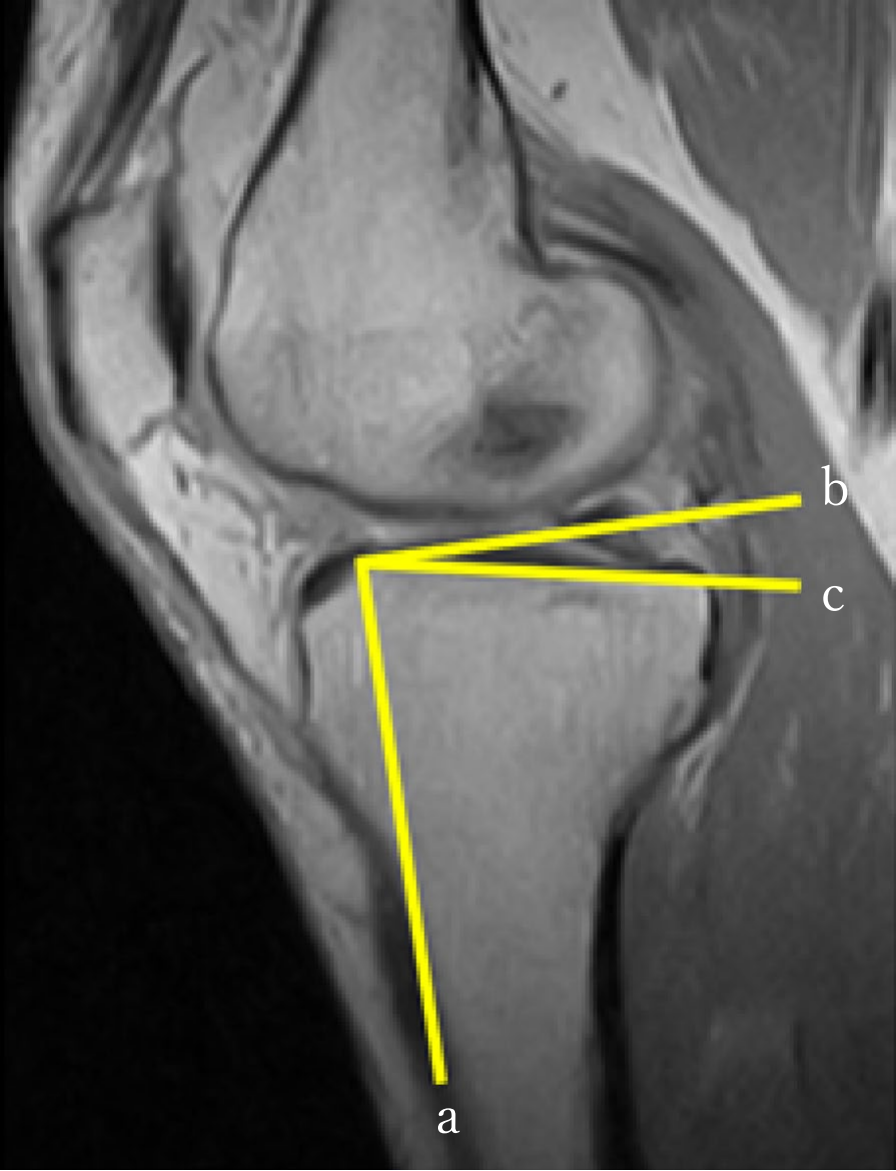
Measurement of medial tibial posterior slope (MTPS). **a**: Parallel line of the diaphyseal tibial axis in the sagittal slice determined by an axial view of the tibial plateau; **b**: Perpendicular line to the line **a** from the anterior-most point of the tibial joint surface; **c**: Line connecting the posterior-most and anterior-most points; MTPS was determined as the angle between lines **b** and **c**

The MTMS and MTPS were measured according to Hashemi et al. [20] The tibial surface was recognised by the axial view. The longest transverse tibial line in the tibial surface was selected to determine the MTMS in the coronal view. The MTMS was the angle between the perpendicular line of the longitudinal tibial line and the tibial surface line in the coronal view (Fig. 4). The longest longitudinal line of the medial tibial condyle in the tibial surface was selected to determine the MTPS in the sagittal view. The MTPS was the angle between the perpendicular line of the longitudinal tibial line and the tibial medial surface line in the sagittal view (Fig. 5).

The measurements were performed using image processing software (NTD-800b, Hitachi Healthcare Systems Corp., Tokyo, Japan) up to two decimal places.

### Statistical analysis

Statistical analysis was performed using Statcel3(Seiunsha Corp., Japan). The Mann–Whitney U test was used to determine the differences between the two groups for MM degeneration. The Student’s t-test was used for MMME, MMPE, MTMS, and MTPS. Correlation analysis between the degree of MM degeneration and MMME and MMPE was also performed. The *p* value was presented up to three decimal places. Post-hoc power analysis was performed to determine the statistical power of the study using G * Power version 3.1.9.2 (University of Dusseldorf, Germany). The power analysis using post-hoc analysis determined 0.79 when the effect size was 0.5 and α error was 5%.

The inter-observer and intra-observer reliabilities were assessed with the intra-class correlation coefficient (ICC) with regard to meniscus degeneration grade (0–4), MMME, MMPE, MTMS, and MTPS using randomly selected 20 MRIs. The inter-observer reproducibility and intra-observer repeatability were 0.95 and 0.92 for meniscus degeneration grade, 0.99 and 0.98 for MMME, 0.98 and 0.98 for MMPE, 0.95 and 0.95 for MTMS, and 0.96 and 0.97 for MTPS, respectively. Every ICC showed high measurement reliability in this study.

## Results

### Medial meniscus degeneration

The MM degeneration grade in the anterior portion of the MCL averaged 1.72 (SD: standard deviation, 0.67) in the PRT group and 1.40 (SD 0.78) in the non-PRT group. The MM degeneration was statistically significantly larger in the PRT group (*p* = 0.050). The differences of MM degeneration were not statistical in the other portions of the middle MCL, posterior to MCL, and mid-posterior segment (Table 2). The meniscus degeneration grades in each portion for both groups are shown in Table 3.

**Table 2:** Medial meniscus degeneration grade (average ± SD) according to Jerosch et al. ^17) ^at 4 portions

group portion	MMPRT +	MMPRT-	*p*-value
Anteromedial	1.72 ± 0.67	1.40 ± 0.78	0.050
Medial	2.29 ± 0.68	2.11 ± 0.89	0.637
Posteromedial	2.73 ± 0.79	2.80 ± 0.84	0.668
Posterior	2.56 ± 0.65	2.67 ± 0.73	0.508

MMPRT: medial meniscus posterior root tear,

The *p*-value determined by the Mann–Whitney U test was presented up to three decimal places

**Table 3:** Distribution of MM degenerative grade at each 4 portion in MMPRT + and MMPRT-

	MMPRT +					MMPRT-				
Degeneration grading (0-4^a^) Portion	0%	1	2	3	4	0	1	2	3	4
Anteromedial	6.3	20.8	66.7	6.3	0	18.4	22.3	59.2	0	0
Medial	0	4.2	70.8	16.7	8.3	7.8	6.8	55.3	25.2	3.9
Posteromedial	0	0	47.9	31.3	20.8	0	0	45.6	26.2	27.2
Posterior	0	0	52.1	39.6	8.3	0	0	48.5	35.0	15.5

*MM* medial meniscus, *MMME* medial meniscus medial extrusion, *MMPE* medial meniscus posterior extrusion

^a^Meniscus degeneration graded according Jerosch et al. ^17)^

### Meniscus extrusion

The MMME averaged 4.02 mm (SD 1.12) in the PRT group and 3.11 mm (SD 1.11) in the non-PRT group. The MMME of the PRT group was statistically significantly greater than that of the non-PRT group (*p* < 0.001). The MMPE averaged 4.22 mm (SD 0.87) in the PRT group and 2.83 mm (SD 1.12) in the non-PRT group. The MMPE of the PRT group was statistically significantly greater than that of the non-PRT group (*p* < 0.001).

### Correlation between MM degeneration and meniscus extrusion

For all knees (*n* = 151), MMME and MMPE were shown at each degree of MM degeneration (Table 4). The correlation coefficient between the degree of MM degeneration and MMME was 0.93 (*p* < 0.001). The correlation coefficient between the degree of MM degeneration and MMPE was 0.81 (*p* = 0.001).

**Table 4:** Distribution of degree of MM degeneration, and medial and posterior extrusion in all knees

Degree of MM degeneration	Number of knees	MMME (mm)	MMPE (mm)
4	4	2.53 ± 1.08	2.01 ± 1.04
5	5	1.97 ± 0.95	2.69 ± 1.65
6	14	2.81 ± 0.93	3.12 ± 1.49
7	15	3.31 ± 1.21	3.14 ± 0.94
8	31	3.24 ± 1.19	3.34 ± 1.36
9	22	3.06 ± 1.03	3.16 ± 1.30
10	17	3.70 ± 1.13	3.03 ± 0.88
11	8	3.85 ± 0.49	3.91 ± 0.49
12	20	3.93 ± 1.10	3.65 ± 1.06
13	11	3.80 ± 0.87	3.01 ± 1.05
14	3	4.35 ± 1.98	4.16 ± 0.97
15	1	5.15	4.04

*MMME* medial meniscus medial extrusion

*MMPE* medial meniscus posterior extrusion

For knees without PRT (*n* = 103), the correlation coefficient between the degree of MM degeneration and MMME was 0.82 (*p* = 0.002). The correlation coefficient between the degree of MM degeneration and MMPE was 0.82 (*p* = 0.002).

For knees with PRT (*n* = 48), the correlation coefficient between the degree of MM degeneration and MMPE was -0.05 (*p* = 0.892). The correlation coefficient between the degree of MM degeneration and MMPE was -0.32 (*p* = 0.344).

### Medial tibial slope

The MTMS averaged 3.65° (SD 1.54) in the PRT group and 3.57° (SD 1.45) in the non-PRT group. The MTMS showed no statistical difference between the two groups (*P* = 0.754). The MTPS averaged 6.34° (SD 2.25) in the PRT group and 5.28° (SD 1.11) in the non-PRT group. The MTPS of the PRT group was statistically significantly greater than that of the non-PRT group (*p* = 0.007).

## Discussion

The most significant findings of this study were that the medial-type knee OA with PRT showed a statistically significantly progressed MM degenerative change in the anterior portion, greater anterior and posterior MM extrusion, and tibial posterior slope compared to those without PRT.

A new finding from this MRI study will be only in the evaluation of MM degeneration. Each other finding has been already reported separately. General assessment of the knee will be important in respect to tibial morphology, degeneration and extrusion of the MM in the treatment. The current study contributes to better assessment of the MRI of osteoarthritic knees.

Jones et al. [21] showed that the anterior segment of the MM bears an average of 2.86% greater hoop stress than the posterior segment compared to the average of 1.54% increase in an in-vitro study using cadaveric knees. LaPrade et al. [4] reported that the medial contact stress increased by an average of 106% towards the anteromedial joint surface. The results of this study revealed increased MM degeneration anteriorly with greater anterior extrusion, which means that decreased hoop stress caused the increasing load on the anteromedial joint surface in the PRT group.

Okazaki et al. [22] reported that the MMME averaged 3.4 ± 0.7 (SD) mm in the PRT group and 1.4 ± 0.6 mm in the normal control and MMPE averaged 3.4 ± 1.3 mm in the PRT group and 1.3 ± 0.8 mm in the normal control. Both the MMME and MMPE were statistically significantly greater in the PRT group. Another study by Okazaki et al. [23] showed that MTPS averaged 4.0 ± 1.9° (SD) in the ACL injured group, 3.5 ± 1.4° in the volunteer group, and 7.2 ± 1.9° in the MMPRT group. The MTPS of the MMPRT group was statistically significantly greater than those of the ACL injury and volunteer groups. The OA knee with PRT featured greater MTPS morphologically, which may have increased the stress on the MM causing its extensive degeneration.

The correlation analysis of this study revealed a strong correlation between the extrusion and degeneration grades of the MM in osteoarthritic knees. There was no such correlation in the knees with PRT. Therefore, the rapid progression of MM extrusion may occur after the posterior root tear occurs despite of the degree of MM degeneration.

Recently, the aim of PRT repair has been restoring the MM function. However, the efficacy of the procedures has not yet reached consensus. Chang et al. [24] reported that pull-out repair could improve the Lysholm and IKDC scores, hospital for special surgery scores, and Tegner scores and could slow the progression of knee OA. However, 49% of patients showed a progression of KL grade in four years on average. They concluded that the progression of knee OA could not be prevented in the medium term by the repair. The present study also raises a question regarding how much the repair of a degenerative meniscus improves MM loading function and how long a repair of a degenerative meniscus maintains MM loading function, as MM was shown to be more degenerative in the PRT group. Though, the repair has the potential to restore MM loading function in the early period after a posterior root tear occurs. The severity of MM degenerative changes should be taken into account when the repair effect is considered. Moreover, as the PRT group showed significantly greater MTPS and reported more MTMS, the morphologic changes should be reconsidered when the operative treatment is planned.

This study has several limitations as a scientific report. First, the study was retrospectively undertaken with a relatively small number of patients, although the power analysis revealed the relatively high power of the study. The time from the onset was not determined, and the body mass index (BMI) was not considered. The onset of MMPRT is not always easy to identify, and the age of the two groups in the present study did not show any significant differences such that a comparison between the two groups was reasonable. Generally, the high BMI of a patient reportedly leads to worse postoperative outcome. The correlation between BMI and MM degeneration should be investigated further. It is also thought that the longer the root tear persists, the more degenerative damage of MM will progress. The tibial slope difference found by this MRI study suggests that the morphologic feature will exist in osteoarthritic knees with PRT. However, actual correction by surgery should be performed primarily according to the long leg anterior–posterior radiograph. Another prospective study with a greater number of patients is advisable in future research.

## Conclusions

The severity of MM degenerative changes, extrusion of MM, and degree of tibial slope were measured between medial-type knee OA with and without PRT using an open MRI. The MM anterior degeneration was more severe in the PRT group. The PRT group showed greater MM extrusion anteriorly and posteriorly with greater posterior tibial slope.

## Data Availability

The datasets used and/or analysed during the current study are available from the corresponding author on reasonable request.
